# In vitro CAR-T cell killing: validation of the potency assay

**DOI:** 10.1007/s00262-024-03753-y

**Published:** 2024-07-02

**Authors:** Claudia Piccinini, Silvia Carloni, Chiara Arienti, Elena Pancisi, Francesca Fanini, Sara Pignatta, Valentina Soldati, Monica Stefanelli, Anna Maria Granato, Giovanni Martinelli, Laura Ridolfi, Massimiliano Petrini

**Affiliations:** 1 Present Address: Immuno-Gene Therapy Factory, IRCCS Istituto Romagnolo per lo Studio dei Tumori (IRST) “Dino Amadori”, Via Maroncelli 40, 47014 Meldola, Italy; 2 Experimental and Clinical Oncology of Immunotherapy and Rare Cancer Unit, IRCCS Istituto Romagnolo per lo Studio dei Tumori (IRST) “Dino Amadori”, Meldola, Italy; 3 Scientific Direction, IRCCS Istituto Romagnolo per lo Studio dei Tumori (IRST) “Dino Amadori”, Meldola, Italy

**Keywords:** Potency, CAR-T, Killing, Validation

## Abstract

For advanced therapy medicinal products, the development and validation of potency assays are required, in accordance with international guidelines, to characterise the product and obtain reliable and consistent data. Our purpose was to validate the killing assay for the evaluation of autologous anti-CD19 chimeric antigen receptor (CAR) T potency. We used CD4 + and CD8 + lymphocytes or anti-CD19 CAR-T cells as effector cells and REH (CD19 +) or MOLM-13 (CD19 −) cell lines as target cells. After co-culturing target and effector cells (1:1 ratio) for 24 h, samples were labelled with 7-AAD, anti-CD3 and anti-CD19 antibodies and the frequency of CD19 + dead cells was evaluated by flow cytometry. In order to verify the CAR-T specificity for the CD19 + target, the co-culture between CAR-T and REH or MOLM-13 at different effector-to-target ratios was scheduled. Moreover, not transduced CD4 + and CD8 + lymphocytes were tested in comparison with CAR-T from the same donor to demonstrate the assay specificity. Linearity and accuracy were evaluated, and established acceptance criteria were compiled for both parameters (r^2^ ≥ 0.97 for linearity and average relative error ≤ 10% for accuracy). Furthermore, the method was considered robust when performed between 23 and 25 h of co-culture, and the intra-assay, inter-assay and inter-day precision was obtained. Finally, in order to verify the inter-analyst precision, the test was executed by three different operators and the intra-class correlation coefficient was > 0.4 in both cases. In conclusion, we consider this CAR-T potency assay as validated and usable in all steps of product development and quality control.

**Supplementary Information:**

The online version contains supplementary material available at 10.1007/s00262-024-03753-y.

## Introduction

Advanced therapy medicinal products (ATMPs) are complex biological products that require in-depth characterisation to ensure their quality, safety and efficacy [1, 2]. To properly understand the functionality of the product, it is essential to perform potency assays that should be based on its mechanism of action and sensitive enough to detect changes that may affect its activity and function [3, 4]. The Food and Drug Administration (FDA) regulations allow for considerable flexibility in determining the appropriate potency measurements for each ATMP, but require compliance with current good manufacturing practice (cGMP) for all potency assays used for release testing [5]. Among the genetically engineered ATMPs, one promising treatment is based on chimeric antigen receptor (CAR) T cells, a product derived from modified autologous T cells able to recognise specific antigens and killing tumour cells [6, 7]. The efficacy of CAR-T cells depends on different cell functions, such as cytotoxic activity, which could be measured by potency assay [8]. In general, cytotoxicity assays involve co-culture of target and effector cells to mimic in vivo cell function. There are various outputs to measure the cytotoxic effect, such as bioluminescence and impedance [9, 10]. Alternatively, using vital dyes, flow cytometry is also a sensitive method for detecting target cell death. Moreover, co-culture times and cell ratios are highly variable, depending on the type of cell involved and the output chosen. For all these reasons, the establishment of a standard potency assay protocol is tricky [11, 12] and method validation is required, including at least analysis of specificity, linearity, accuracy, robustness and precision [4, 13, 14].

The aim of this study was to develop and validate a cytofluorimetric cytotoxicity assay able to quantify the potency of cGMP-manufactured anti-CD19 CAR-T cells.

## Materials and methods

### Cell culture

The human REH cell line was purchased from the American Type Culture Collection (Rockville, MD, RRID:CVCL_1650), while the human MOLM-13 cell line was obtained from DSMZ-German Collection of Microorganisms and Cell Cultures (Braunschweig, Germany, RRID:CVCL_2119). Genetic characteristics of cell lines were determined by PCR-single-locus-technology with 16 independent PCR-systems (Eurofins Genomics, Ebersberg, Germany). Both cell lines were maintained at 37 °C and 5% CO_2_ in RPMI (Euroclone, Milan, Italy) supplemented with 10% foetal bovine serum (FBS; Gibco-Thermo Fisher Scientific, Waltham, MA, USA) and used for experiments up to the fifteenth subculture passage. Cells were screened for mycoplasma contamination using the MycoAlert Mycoplasma Detection Kit (Lonza Bioscience-Euroclone, Milan, Italy) according to the manufacturer’s instructions.

### CAR-T manufacturing

After obtaining the donor’s informed consent in accordance with the Declaration of Helsinki, buffy coats were used for CAR-T production and processed in the CliniMACS Prodigy closed system (Miltenyi Biotec), as previously described [15]. CD4 + and CD8 + lymphocytes were isolated by immunomagnetic selection using CliniMACS CD4 and CD8 Reagent (Miltenyi Biotec Cat# 200-070-132, Cat# 200–070-115) and cultured in TexMACS medium (Miltenyi Biotec Cat# 170-076-306) supplemented with MACS GMP recombinant human interleukin (IL)-7 and IL-15 (Miltenyi Biotec Cat# 170-076-111, Cat# 170-076-114). To transduce the selected cells, the lentiviral vector CD19 CAR SF (Miltenyi Biotec Cat# 200-072-102) was added to the cell culture. On day 12, CAR-T cells were collected. CD4 + and CD8 + lymphocytes and CAR-T cells were stored in nitrogen vapour in 90% heat-inactivated human serum AB (Biowest, Nuaillé, France) and 10% dimethyl sulfoxide (Mylan, Dublin, Ireland) solution until use. Cryopreserved samples were thawed, washed and resuspended in RPMI and 10% of FBS before use.

### Immunophenotypic analysis

REH and MOLM-13 cells (3 × 10^5^) were analysed for CD19 expression prior to use by staining with anti-CD19 REA Antibody APC-Vio770 1:50 (Miltenyi Biotec Cat# 130-113-643, RRID:AB_2726196) for 10 min at 4 °C in the dark. Appropriate conjugated REA Controls (S) (1:50; Miltenyi Biotec) were included for each sample. Only REH expressing CD19 levels > 70% or MOLM-13 negative for CD19 expression were considered useful for further analysis.

The immunophenotype of CAR-T cells (3–10 × 10^5^) was performed with Stain Express CART-T Transduction Cocktail (Miltenyi Biotec Cat# 130-127-638) with anti-CD19 CAR FMC63 Idiotype Antibody PE 1:50 (Miltenyi Biotec Cat# 130-127-342), incubated for 10 min at room temperature in the dark and then acquired to the flow cytometer.

### Killing assay

The cytotoxic ability of CAR-T cells was evaluated using effector cells derived from the manufacturing process (CD4 + and CD8 + lymphocytes and CAR-T cells) and REH cells as the target of the cytotoxic effect. For the exclusion of dead cells, a flow cytometric analysis was performed before seeding using 7-amino-actinomycin D (7-AAD) staining solution (Miltenyi Biotec Cat# 170–080-032), according to the manufacturer's instructions. The assay was performed in a 24-well plate by co-culturing 7-AAD- CD4 + and CD8 + lymphocytes (background) or CAR-T cells (killing) and target cells in a 1:1 ratio for 24 h, considering the percentage of CAR-T transfection. A negative control (CTR −) of REH cells only was included in each plate. Then, cells were stained for 10 min at 4 °C in the dark with anti-CD3 Viogreen (Miltenyi Biotec Cat# 130–113-142, RRID:AB_2725970) and anti-CD19 APC-Vio770 REA Antibodies 1:50 and 7-AAD to determine the frequency of dead target cells by flow cytometry.

### Flow cytometry

Flow cytometry acquisition was carried out on the MACSQuant Analyzer 10 (Miltenyi Biotec) equipped with 405 (violet), 488 nm (blue) and 640 (red) lasers and 10,000 events were recorded for each sample. The acquisition and analysis gates were set on forward and side scatter properties of cells. Flow cytometry data were analysed with MACSQuantify Software version 2.13.2 with Express Mode version 213.6.20466 (Miltenyi Biotec).

### Data and statistical analysis

The potency was calculated by subtracting the mortality rate (7-AAD + events) of the background well (CD4 + and CD8 + lymphocytes and REH) from that of the sample under test (CAR-T and REH). The coefficient of variation (CV%) was used to describe the variability of potency results obtained throughout the method validation. Data obtained from different analysts were compared using the intra-class correlation coefficient (ICC) based on absolute agreement, two-way mixed-effect model. For specificity assessment, differences in death induced by CAR-T and CD4 + and CD8 + cells were determined using the Student’s *t*-test for unpaired observation. *P* < 0.05 was considered statistically significant. All statistical analyses were performed using SPSS 22.0.

## Results

### Method development

**Fig. 1:**
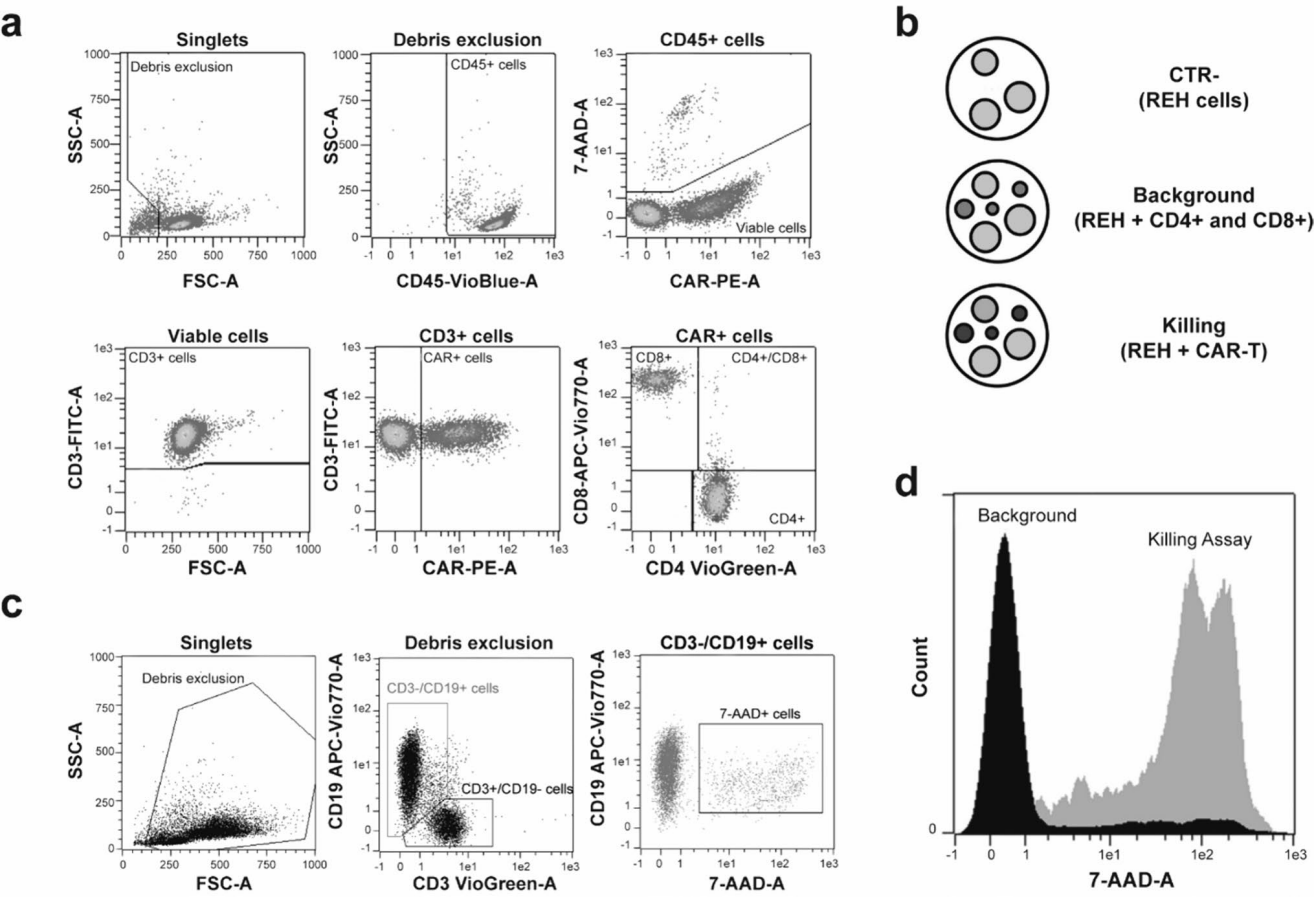
Experimental design and gating strategy. **a** Gating strategy to quantify and characterise CAR-T cells. **b** Seeding conditions for the killing test. **c** Representative flow cytometric dot plots containing the gating strategy used to identify dead target cells. REH cells were gated based on their CD3 − /CD19 + phenotype, while effector cells were CD3 + /CD19 − . Dead target cells were assessed by selecting CD3 − /CD19 + /7-AAD + cells. **d** The histogram shows the overlay of background and killing conditions. All the flow cytometric analyses were performed excluding doublets (not shown)

Four batches of CAR-T cells were initially immunophenotyped (Fig. 1a) in accordance with guidelines [16]. The flow cytometric analysis showed that the percentage of viable CD3 + cells was always above 90% (mean ± standard deviation (SD): 99.13 ± 0.61%), while the average percentage of viable CD3 + /CAR + cells was 36.33 ± 6.09%. The obtained results meet the specifications (CD3 +  ≥ 90% and CD3 + /CAR +  ≥ 20%).

In order to assess the best experimental conditions, we evaluated several effector to-target ratios (*E*:*T*) and three different incubation times (6–24–48 h). The CD4 + and CD8 + cells in the finished product may have an intrinsic cytotoxic activity, and therefore, they have been included as effectors (Fig. 1b). The frequency of dead target cells was quantified as CD3 − /CD19 + /7-AAD + (Fig. 1c–d).

**Fig. 2:**
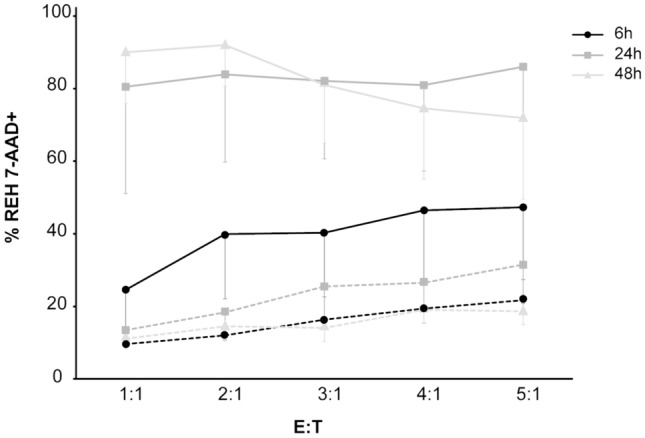
Dead target cells at different incubation times and *E*:*T* ratios. Mean values obtained after co-culture of REH cells with three batches of CAR-T (continuous lines) or CD4 + and CD8 + cells (dashed lines) were reported. Error bars represent the SD

Our results showed that high concentrations of effector cells during the longest co-culture time caused excessive cell death to allow for a proper flow cytometric evaluation, whereas, after 6 h, the observed cytotoxic effect was not appreciably different from the background (Fig. 2). Hence, we decided to carry out all further co-cultures for a period of 24 h.

### Method validation

The validation of the method described in this paper was performed in compliance with the essential analytical parameters defined by the Guidelines of the International Conference of Harmonization [4] and in accordance with the US FDA guidance document [14].

### Specificity

**Fig. 3:**
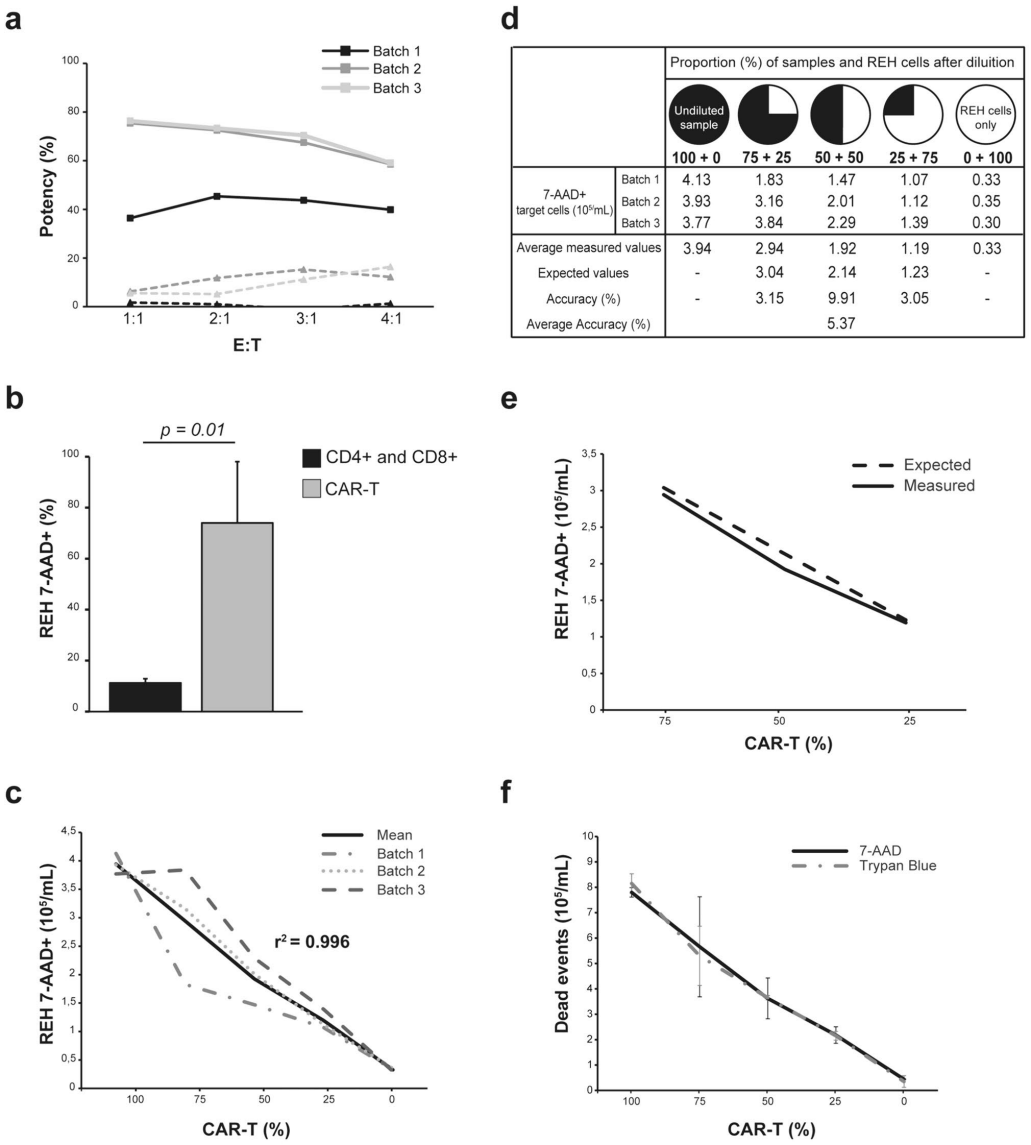
Assay validation. **a** Potency induced by different ratios of three batches of CAR-T and target cells. Continuous lines represent the effect on REH cells; the dashed lines represent the effect on MOLM-13 cells. **b** Histograms represent the mean and SD of target cell death (%) determined by three batches of CD4 + and CD8 + cells and CAR-T cells. **c** Number of dead target cells obtained by different dilutions of the killing condition. The *x*-axis shows the dilution (%) with the live REH cells of the CAR-T + REH well. Dashed lines represent three CAR-T batches, and the continuous line represents the calculated mean value. Acceptance criterion: *r*^2^ ≥ 0.97. **d** Relation between expected and average measured values expressed as number of dead target cells induced by three different batches of CAR-T. The expected death value was calculated as the multiple of the proportion of sample after dilution and the average measured value obtained from the undiluted sample, plus the multiple of the proportion of added REH and the average measured value obtained from the REH cells only. Acceptance criterion: average accuracy ≤ 10%. **e** Comparison between the calculated expected value of the target cell death and the measured value. **f** Dead events measured with flow cytometry (black continuous line) and trypan blue staining (grey dashed line). To allow comparison between flow cytometric analysis and light microscopic counting, we considered all 7-AAD + events. Error bars represent the SD

Different concentrations of effector cells were co-cultured with REH or MOLM-13 cells. The aim of the experiment was to demonstrate that anti-CD19 CAR-T cells induced cell death only in CD19 + target cells. As we expected, the potency appeared definitely lower in CD19 − compared to CD19 + cell line, respecting the established acceptance criteria, thus defining the method as specific (Fig. 3a; Supplementary Table 1). Furthermore, when cells were seeded at a 1:1 ratio, the potency of all tested CAR-T batches on MOLM-13 became negligible and the highest mean percentage change was obtained. For this reason, the 1:1 ratio was maintained throughout the subsequent experiments. In addition, cytotoxic activity was assessed with three different batches of CAR-T or CD4 + and CD8 + cells to demonstrate that REH cell death was specifically induced by CAR-T cells rather than non-transduced CD4 + and CD8 + . As shown in Fig. 3b, cell death induced by CAR-T cells was significantly higher than that induced by CD4 + and CD8 + cells (*p* = 0.01).

### Linearity

The aim of the experiment to demonstrate the proportionality between the concentration of CAR-T cells seeded and the target cell death was achieved. At the end of the co-culture time at 1:1 ratio, samples were serially diluted with live REH cells and stained according to the killing assay. The obtained *r*^2^ (Fig. 3c) indicated that our method produced results directly proportional to the concentration of dead target.

### Accuracy

Due to the inherent nature of our product, it was not possible to define a true value, but rather the agreement between the obtained cell death values and those expected. To demonstrate the accuracy of the method, the experiment was carried out as described in the previous section, diluting after co-culture the obtained samples with live REH cells. The expected value was determined by the contribution exerted by the quantity of CAR-T and REH cells present in the co-culture (Fig. 3d–e). In addition, cell death was assessed using the method of trypan blue viable counting to provide an additional expected value against which to compare the data obtained (Fig. 3f). The average accuracy was 5.76% and in both cases met the established acceptance criteria, defining the method as accurate.

### Robustness

The purpose of the test was to evaluate the results after 23, 24 and 25 h of incubation times on three different batches of CAR-T cells. The CV obtained was always ≤ 10% and the method was considered robust at the experimental times between 23 and 25 h of co-culture (Supplementary Table 2).

### Precision

The intra-assay precision was evaluated in triplicate on a batch of CAR-T cells to determine the well-to-well variation. The evaluation of intermediate precision included three runs on the same day by the same operator (inter-assay), three runs on different days by the same operator (inter-day), and three runs by different operators (inter-analyst). For all of the above tests, the precision remained within the established acceptance criteria (Table 1).

**Table 1:** Repeatability and intermediate precision of killing assay

	Acceptance criteria	Results
Intra-assay	CV = 10%	CV = 3.13%
Inter-assay	CV = 20%	CV = 2.78%
Inter-day	CV = 20%	CV = 14.82%
Inter-analyst 1	ICC^a^ > 0.4	0.94
Inter-analyst 2	ICC^a^ > 0.4	0.95

^a^Based on the 95% confidence interval of the ICC estimate, values less than 0.5, between 0.5 and 0.75, between 0.75 and 0.9, and greater than 0.90 are indicative of poor, moderate, good, and excellent reliability, respectively

### Discussion

A CAR-T cell-based ATMP is a customised therapy designed to treat mainly haematological tumours with a relevant clinical response. The high efficacy of the therapy, the variability of the manufacturing process and the serious side effects highlight the need for appropriate quality control of CAR-T cell therapies [6, 8, 17]. In order to ensure the safety, identity, quality and purity, a comprehensive analysis of ATMPs is mandatory. In particular, international guidelines recommend a matrix approach to measure potency. The inclusion of potency analysis in routine release tests is advisable, but the high variability of products and the low standardisation of the assay make it difficult to apply. In addition, the use of a potency assay for release of a production batch requires careful method validation [12]. Our aim was to develop and validate a flow cytometric potency assay to evaluate the cytotoxic activity of CAR-T therapy. The obtained results validated our killing assay when performed under the specified tested conditions. In particular, we demonstrated that the test was able to specifically quantify the cell death induced by anti-CD19 CAR-T on REH cells without interference from non-target (CD19 −) cells and non-effector (not transduced) cells. Moreover, death quantification was directly proportional to the concentration of CAR-T cells in the sample and in accordance with the true value in terms of calculated expected values, or values obtained with trypan blue staining. Finally, the test showed acceptable levels of robustness and precision, and it could be easily performed by different analysts while maintaining reproducibility. These features made our validated potency assay easily scalable and therefore ideal for process validation and release analysis. In addition, the proposed assay is free of method-related health hazards and allows the identification of dead target cells. The validation of this CAR-T assay potency allows the method to be used in all steps of ATMP development and quality control.

### Supplementary Information

Below is the link to the electronic supplementary material.

Supplementary file1 (DOCX 13 kb)

## Data Availability

Raw data were generated at Immuno-Gene Therapy Factory. Derived data supporting the findings of this study are available from the corresponding author SP on reasonable request.
