# Supplementary material for: In vitro CAR-T cell killing: validation of the potency assay
Source: Cancer Immunol Immunother. 2024 Jul 2;73(9):168. doi: 10.1007/s00262-024-03753-y (PMC11219661; doi:10.1007/s00262-024-03753-y)
Supplement: Supplementary file 1 — Supplementary file1 (DOCX 13 kb) [file 262_2024_3753_MOESM1_ESM.docx]

**Table 1. Specificity as divergence of potency obtained on different target cells.**

|  | **Percentage change (%)** | | | |
| --- | --- | --- | --- | --- |
|  | **1:1** | **2:1** | **3:1** | **4:1** |
| **Batch 1** | 95.3 | 97.8 | 102.5 | 96.8 |
| **Batch 2** | 91.7 | 83.7 | 77.3 | 79.2 |
| **Batch 3** | 92.7 | 92.9 | 84.1 | 72.3 |
| **Mean^a^** | 93.2 | 91.5 | 88.0 | 82.8 |
| **SD** | 1.86 | 7.16 | 13.04 | 12.63 |

*^a^Acceptance criterion: percentage change between REH and MOLM-13 potency values > 70%*

**Table 2. Evaluation of potency at different co-culture times.**

| **Potency (%)** | | | |
| --- | --- | --- | --- |
|  | **Batch 1** | **Batch 2** | **Batch 3** |
| **23h** | 73,24 | 74,14 | 74,44 |
| **24h** | 83,83 | 84,32 | 84,07 |
| **25h** | 86,95 | 87,65 | 88,9 |
| **Average** | 81,34 | 82,04 | 82,47 |
| **SD** | 7,19 | 7,04 | 7,36 |
| **CV (%)** | 8,83 | 8,58 | 8,93 |
